# Genetic variants of the autophagy pathway as prognostic indicators for prostate cancer

**DOI:** 10.1038/srep14045

**Published:** 2015-09-14

**Authors:** Chao-Yuan Huang, Shu-Pin Huang, Victor C. Lin, Chia-Cheng Yu, Ta-Yuan Chang, Te-Ling Lu, Hung-Chih Chiang, Bo-Ying Bao

**Affiliations:** 1 Department of Urology, National Taiwan University Hospital, College of Medicine, National Taiwan University, Taipei, Taiwan; 2 Department of Urology, Kaohsiung Medical University Hospital, Kaohsiung, Taiwan; 3 Department of Urology, Faculty of Medicine, College of Medicine, Kaohsiung Medical University, Kaohsiung, Taiwan; 4 Department of Urology, E-Da Hospital, Kaohsiung, Taiwan; 5 School of Medicine for International Students, I-Shou University, Kaohsiung, Taiwan; 6 Division of Urology, Department of Surgery, Kaohsiung Veterans General Hospital, Kaohsiung, Taiwan; 7 Department of Urology, School of Medicine, National Yang-Ming University, Taipei, Taiwan; 8 Department of Pharmacy, Tajen University, Pingtung, Taiwan; 9 Department of Occupational Safety and Health, China Medical University, Taichung, Taiwan; 10 Department of Pharmacy, China Medical University, Taichung, Taiwan; 11 Sex Hormone Research Center, China Medical University Hospital, Taichung, Taiwan; 12 Department of Nursing, Asia University, Taichung, Taiwan

## Abstract

Autophagy is a complex process of autodigestion in conditions of cellular stress, and it might play an important role in the pathophysiology during carcinogenesis. We hypothesize that genetic variants of the autophagy pathway may influence clinical outcomes in prostate cancer patients. We genotyped 40 tagging single-nucleotide polymorphisms (SNPs) from 7 core autophagy pathway genes in 458 localized prostate cancer patients. Multivariate Cox regression was performed to evaluate the independent association of each SNP with disease progression. Positive findings were then replicated in an independent cohort of 504 advanced prostate cancer patients. After adjusting for known clinicopathologic factors, the association between *ATG16L1* rs78835907 and recurrence in localized disease [hazard ratio (HR) 0.70, 95% confidence interval (CI) 0.54–0.90, *P* = 0.006] was replicated in more advanced disease (HR 0.78, 95% CI 0.64–0.95, *P* = 0.014). Additional integrated *in silico* analysis suggests that rs78835907 tends to affect *ATG16L1* expression, which in turn is correlated with tumor aggressiveness and patient prognosis. In conclusion, genetic variants of the autophagy pathway contribute to the variable outcomes in prostate cancer, and discovery of these novel biomarkers might help stratify patients according to their risk of disease progression.

Prostate cancer is one of the most commonly diagnosed cancers in men. The incidence of prostate cancer is on the increase probably owing to widespread availability of serum prostate-specific antigen (PSA) testing, leading to increased detection of localized prostate cancer^1^. Radical prostatectomy (RP) is widely performed as the definitive treatment for localized prostate cancer. Although traditional prognostic factors, including PSA levels, tumor stage, Gleason score, and surgical margin status, are often used to predict outcomes after RP, biochemical recurrence (BCR) has still been shown to occur in 20–40% of patients within 10 years^2^. It has been estimated that approximately 20% of emerging prostate cancers are locally advanced^3^, and androgen deprivation therapy (ADT) constitutes the first-line treatment for most of these patients. Despite frequent good outcomes of this therapy, many patients eventually develop castration-resistant prostate cancer and distant metastasis, accounting for the majority of the mortality from the prostate cancer^4^. As the efficacy of prostate cancer treatments is highly variable, there is a need to identify additional biomarkers to improve outcome prediction and tailor individual therapeutic interventions.

Autophagy is a homeostatic process that involves lysosomal degradation of cytoplasmic components, and is now widely implicated in several physiological responses, such as cancer and aging^5^. Evidence supports autophagy as both a tumor suppressor and a tumor promoter depending on the cancer type, stage, and therapy context^6^. It has been hypothesized that autophagy provides an anticarcinogenic function by safeguarding cellular integrity against metabolic stress through the homeostatic turnover of damaged organelles and the clearance of protein aggregates. However, autophagy may also confer a survival advantage on tumor cells that are under metabolic stress, such as hypoxia from insufficient vascularization and selective pressure from therapeutic interventions. The core autophagy machinery in mammals can be divided into 3 functional groups: (i) the induction complex, involving unc-51 like autophagy activating kinase 1 (ULK1), for the formation of a preautophagosomal structure in response to signals; (ii) mediators of autophagosome nucleation, involving beclin 1 (BECN1), for the generation of phosphatidylinositol-3-phosphate and promotion of autophagosomal membrane nucleation; (iii) mediators of autophagosome elongation, involving autophagy related 12 (ATG12) and microtubule-associated protein 1 light chain 3 (MAP1LC3) and their conjugation machinery (ATG5 and ATG16L1), for assisting in the elongation of the autophagic membrane. The protein sequestosome 1 (SQSTM1), also known as p62, contains domains to interact with ubiquitinated proteins and the MAP1LC3, acting as adaptors between the autophagic machinery and the ubiquitinated substrates, which will be selectively eliminated by autophagy. Recent clinical studies have associated aggressive tumor phenotypes with aberrant expression of MAP1LC3, BECN1, and SQSTM1 in prostate cancer^7^, but more number of studies are needed to unravel the complex relationships between autophagy and outcomes after cancer treatment.

In this study, we evaluated the influence of genetic variants within the autophagy pathway on disease progression in localized prostate cancers, and then replicated the findings in locally advanced prostate cancers.

## Results

### Characteristics of the participants

The basic characteristics of patients with prostate cancer are shown in Table 1. For localized prostate cancer cohort, we observed 184 (40%) patients with BCR during a median follow-up period of 54 months. PSA level, pathologic Gleason score, and tumor stage affected the recurrence rate (*P* < 0.001). For advanced prostate cancer cohort, 457 (91%) patients showed disease progression with a median follow-up time of 87 months. Demographic features such as age, PSA at ADT initiation, Gleason score, tumor stage, PSA nadir, time to PSA nadir, and treatment modality were significantly associated with disease progression (*P* ≤ 0.022).

### Association of autophagy pathway single-nucleotide polymorphisms (SNPs) with prostate cancer outcomes

**Figure 1:**
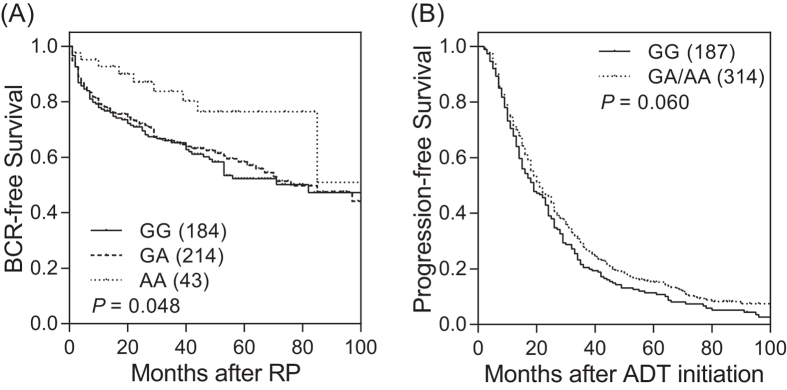
Impact of *ATG16L1* rs78835907 on prostate cancer progression. Kaplan-Meier estimates of (**A**) BCR-free survival in localized prostate cancer patients who underwent RP, and (**B**) progression-free survival in advanced prostate cancer patients who received ADT, by *ATG16L1* rs78835907 genotypes. Numbers in parentheses indicate the number of patients.

Of the 40 SNPs we analyzed from 7 major autophagic genes, 9 SNPs showed nominal associations with BCR after making adjustments for known clinicopathologic variables in localized prostate cancer patients (*P* < 0.05, Supplementary Table S1). We minimized the false discovery by using a false-discovery rate (FDR) of 10% (*q* < 0.10). Three SNPs, *ATG16L1* rs78835907, *ATG16L1* rs13021297, and *MAP1LC3B* rs8044820, remained significant (Table 2 and Fig. 1A), and were selected to replicate findings in advanced prostate cancer cohort. Only 1 variant, *ATG16L1* rs78835907, showed significant association with disease progression of advanced prostate cancer after adjustment for known clinical factors (*P* = 0.014, Table 3 and Fig. 1B). Patients with at least one rs78835907 A allele had a 22% reduction in risk of disease progression during the follow-up period (95% confidence interval 0.64–0.95), compared with men with the homozygous wild-type (GG).

### Functional analyses of the *ATG16L1* rs78835907 locus

**Figure 2:**
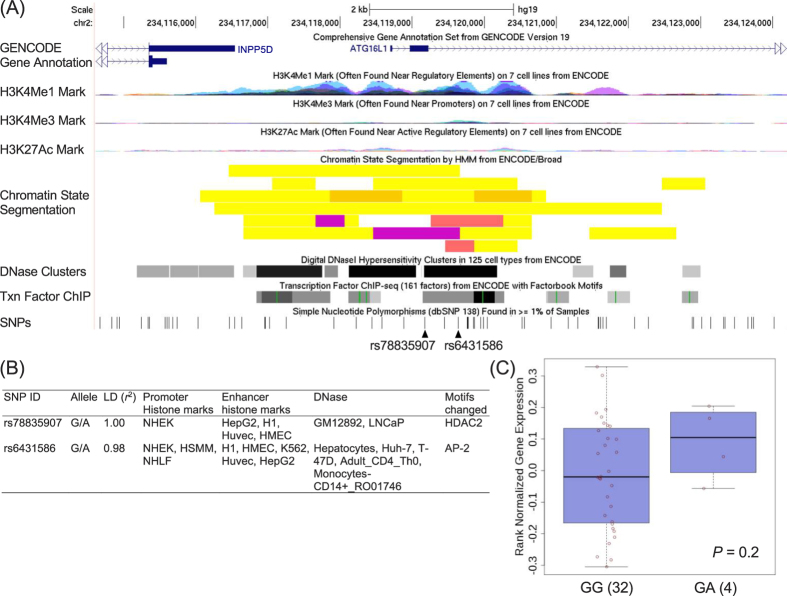
Summary of the functional analyses for the linkage disequilibrium (LD) block containing *ATG16L1* rs78835907. (**A**) Expanded view of the ENCODE data for the LD block containing the *ATG16L1* rs78835907. The H3K4Me1, H3K4Me3, and H3K27Ac tracks show the genome-wide levels of enrichment of the mono-methylation of lysine 4, tri-methylation of lysine 4, and acetylation of lysine 27 of the H3 histone protein, as determined by the ChIP-seq assays. These levels are thought to be associated with promoter and enhancer regions. Chromatin State Segmentation track displays chromatin state segmentations by integrating ChIP-seq data using a Hidden Markov Model for H1 embryonic stem cells, HepG2 hepatocellular carcinoma cells, HUVEC umbilical vein endothelial cells, HMEC mammary epithelial cells, HSMM, skeletal muscle myoblasts, NHEK epidermal keratinocytes, and NHLF lung fibroblasts. The chromatin state regions predicted for promoters and enhancers are highlighted. DNase clusters track shows DNase hypersensitivity areas. Tnx Factor track shows regions of transcription factor binding of DNA, as assayed by ChIP-seq experiments. (**B**) Regulatory annotation of variants within the LD block containing *ATG16L1* rs78835907. In the LD block with the lead SNP rs78835907, ENCODE data showed evidence of promoter and enhancer elements coinciding with the variants in many different cell types. In addition, HDAC2 and AP-2 motifs are predicted to be affected. (**C**) Expression quantitative trait locus association between rs78835907 genotype and *ATG16L1* expression in prostate tissues (GTEx data set). Numbers in parentheses indicate the number of cases.

To identify the putative functional role of rs78835907, functional annotations from the Encyclopedia of DNA Elements (ENCODE) data indicated that rs78835907 and a linked SNP, rs6431586, are situated at a locus with transcription factor binding, DNase hypersensitivity, and histone modification patterns that characterize as promoters or enhancers in several cell types (Fig. 2A). HaploReg also suggested the possible alteration of HDAC2 and AP-2 binding motifs by rs78835907 and rs6431586, respectively (Fig. 2B), indicating that these SNPs might influence gene expression. We then used the Genotype-Tissue Expression (GTEx) database to investigate whether rs78835907 was associated with the expression of *ATG16L1* in human prostate. Individuals carrying a genotype with the variant A at rs78835907 showed a trend of increased expression of *ATG16L1* compared with those with the wild-type homozygous genotype GG, although not statistically significant (*P* = 0.2, Fig. 2C). Interestingly, the effect of rs78835907 on *ATG16L1* expression was consistently observed across various tissue types, including whole blood, skeletal muscle, and another hormone-related tissue, mammary breast (Supplementary Figure S1).

### Correlation of *ATG16L1* expression with prostate cancer progression

**Figure 3:**
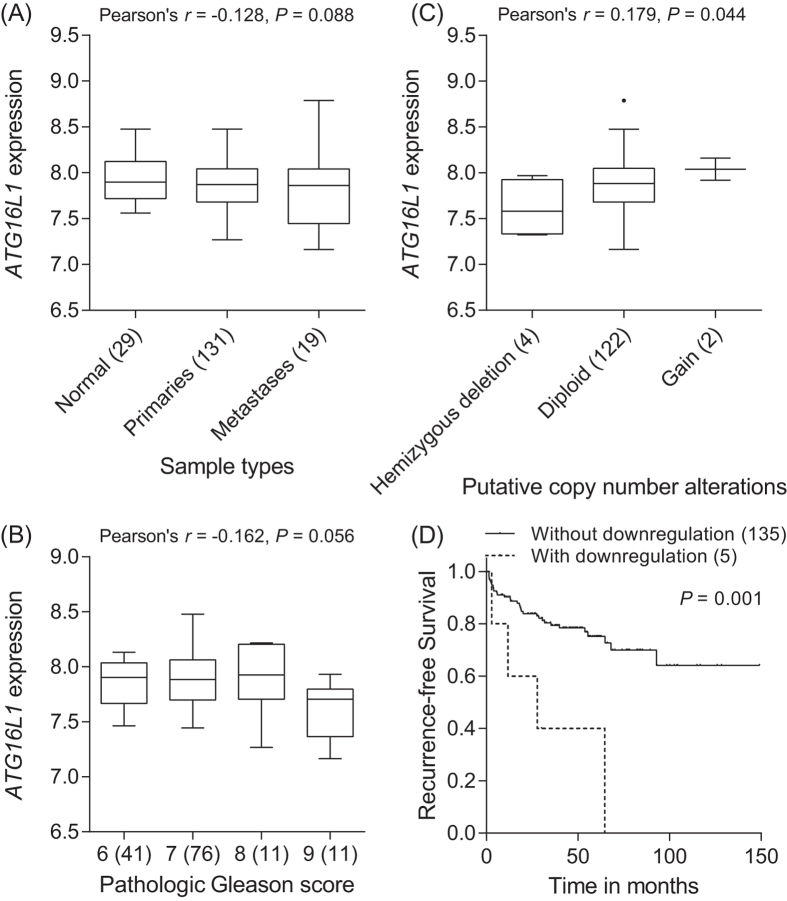
Correlation of *ATG16L1* mRNA expression with prostate cancer progression. The associations between *ATG16L1* expression and prostate cancer aggressiveness were analyzed using MSKCC Prostate Oncogenome data. More advanced prostate cancers with metastasis (**A**) and high pathologic Gleason score (**B**) display a tendency toward lower *ATG16L1* mRNA expression. Numbers in parentheses indicate the number of patients. (**C**) *ATG16L1* shows higher levels of gene expression in tumors with increased DNA copy number at 2q37. (**D**) Kaplan-Meier curves of recurrence-free survival according to the downregulation of *ATG16L1* expression. Patients were dichotomized with or without *ATG16L1* mRNA downregulation (z-scores < −2).

To investigate the association of gene expression levels with prostate cancer outcome, we performed a comprehensive *in silico* analysis using publicly available Memorial Sloan-Kettering Cancer Center (MSKCC) Prostate Oncogenome Project data. There was a trend toward decreased *ATG16L1* gene expression with more aggressive forms of prostate cancer (*P* ≤ 0.088, Fig. 3A,B). Furthermore, gene copy number was found to be correlated with mRNA expression for *ATG16L1* (*P* = 0.044, Fig. 3C). The follow-up of this cohort established that decreased *ATG16L1* expression levels were associated with poorer outcome (*P* = 0.001, Fig. 3D).

## Discussion

We evaluated the association of germline variations in autophagy pathway genes with prostate cancer progression across 2 independent cohorts. *ATG16L1* rs78835907 was consistently associated with a reduced risk of disease progression in patients with localized and advanced prostate cancer. We have also presented additional evidence for a role of ATG16L1 in prostate cancer, as downregulated *ATG16L1* gene expression in tumors was correlated with poorer clinical outcomes, and thereby strengthening the evidence of this genotype-phenotype association.

Recent studies have reported *ATG16L1* polymorphisms were associated with susceptibility of thyroid and colorectal cancers^8^,^9^. However, their influence on disease recurrence and in prostate cancer has not been previously reported. The tagged SNP, rs78835907, in this study is located in the 5′ untranslated region of the *ATG16L1*. Functional annotations from the ENCODE data indicate that rs78835907 and its correlated SNP rs6431586 (*r*^2^ = 0.98) coincide with regions of open chromatin, which probably correspond to the promoters or enhancers of *ATG16L1* (Fig. 2A,B). Specifically, rs78835907 is predicted to lie within a transcription regulatory region, which contains putative binding site for histone deacetylase 2 (HDAC2) that could act as regulators in autophagy progression^10^. Taken together, our data suggest that rs78835907 G allele alters the binding affinity of HDAC2 to the DNA, which in turn reduces the expression of ATG16L1 (Fig. 2C and Supplementary Figure S1), consequently contributing to the more aggressive phenotype and poorer clinical outcome in prostate cancer (Figs 1 and 3). Further biological and functional studies should be accompanied to determine the role of this SNP/gene during prostate cancer progression.

Our findings have potential clinical significance. HDAC inhibitors or inhibitors of lysosomal acidification (e.g. chloroquine and hydroxychloroquine) might be new strategies in the treatment of cancer by modulating autophagy. Current clinical trials are testing whether chloroquine and its derivative, hydroxychloroquine, could enhance chemotherapeutic efficacy (e.g. in the Phase I Trial of MK-2206 and Hydroxychloroquine in Solid Tumors, Melanoma, Renal, and Prostate Cancer [ClinicalTrials.gov Identifier, NCT01480154]).

The strengths of our study are the large number of patients (n = 962) with complete medical information, a systematic coverage of selected autophagy pathway genes, and different stages of the disease. The association of the replicated marker, *ATG16L1* rs78835907, with prostate cancer progression from localized to ADT-treated patients reinforces the validity of our finding. However, the results reported here might be still constrained by multiple comparisons because of the large number of SNPs tested. Although disease progression is a relevant clinical end-point, mortality in prostate cancer patients should be also explored. In addition, our findings from the homogeneous Chinese Han population in this study might be less applicable to other ethnic groups. Therefore, additional large studies with different ethnicity are required to gain further understanding of the contribution of autophagy to prostate cancer biology.

Overall, this study has provided further support for the view that inherited variations may moderate patient outcomes and reveal the importance of autophagy pathway in prostate cancer progression.

## Methods

### Patient recruitment and data collection

Patients diagnosed and confirmed of prostate cancer were recruited from 4 medical centers in Taiwan: National Taiwan University Hospital, Kaohsiung Medical University Hospital, E-Da Hospital, and Kaohsiung Veterans General Hospital, as described previously^11^,^12^,^13^,^14^, and the patients were divided into 2 independent cohorts. The first cohort was composed of 458 patients with localized prostate cancer receiving RP, and the second cohort was composed of 504 patients with advanced disease receiving ADT. Demographic, clinical, and follow-up data were obtained from the medical records. The BCR was defined as 2 consecutive PSA values of at least 0.2 ng/mL after RP^15^,^16^. Disease progression was defined as a serial rise in PSA, at least 2 rises in PSA (>1 week apart), and greater than the PSA nadir while receiving ADT^17^,^18^. Initiation of secondary hormone treatment for rising PSA and deaths from all causes were also considered as progression events. All participants provided written consent, and the local ethics committees approved the research protocol. All the methods applied in the study were carried out in accordance with the approved guidelines.

### SNP selection and genotyping

We utilized a tagging SNP approach to select genetic variants for investigating the genetic variability in the 7 major mammalian autophagic genes, *ATG5*, *ATG12*, *ATG16L1*, *BECN1*, *MAP1LC3B*, *SQSTM1*, and *ULK1*. Tagging SNPs were selected using the Tagger algorithm with *r*^2^ ≥ 0.8, and minor-allele frequencies ≥0.15 based on the 1000 Genomes data for Han Chinese in Beijing, China and Southern Han Chinese^19^. We identified 64 tagging SNPs, which were genotyped at the National Center for Genome Medicine, Taiwan, using the Sequenom iPLEX matrix-assisted laser desorption/ionization time-of-flight mass-spectrometry technology. Genomic DNA was extracted from peripheral blood with the QIAamp DNA Blood Maxi Kit (Qiagen, Valencia, CA, USA) according to the manufacturer’s protocol, and stored until the time of study. For quality control, we randomly selected 10 samples for duplicates, and the concordance rate was >0.99 for all SNPs assayed. Any SNP that failed at Sequenom assay design (n = 17), did not conform to Hardy-Weinberg equilibrium (*P* < 0.01, n = 3), or fell below a genotyping call rate of 0.85 (n = 4), was removed. Thus, a total of 40 SNPs were included for further statistical analyses.

### Statistical analysis

Patient clinicopathologic characteristics were summarized as either the numbers and percentages of patients, or the median and interquartile range of values. The association of clinicopathologic characteristics with time to BCR and disease progression was assessed using the Kaplan-Meier analysis with log-rank test. Multivariate Cox proportional hazards regression analyses were used to assess the effect of each SNP on BCR with adjusting for known prognostic factors, including age, PSA at diagnosis, pathologic Gleason score, and tumor stage, as previously described^12^. We compared 3 genetic models of inheritance to determine the significance of each SNP: dominant (common homozygotes versus variant allele carrying genotypes), recessive (common allele carrying genotypes versus variant homozygotes), and additive (*P* for trend). Only dominant and additive models were considered if the variant homozygotes were observed in <0.05 of the study population. The Statistical Package for the Social Sciences software, version 22.0.0 (IBM, Armonk, NY, USA), was used for statistical analyses. A two-sided *P* value of <0.05 was considered statistically significant. As we were testing 40 SNPs, FDRs (*q* values) were calculated to determine the degree to which the tests for association were prone to false positives using R *q* value package^20^. Associations were deemed significant at the FDR <0.10 level.

### Bioinformatics analysis

We used several bioinformatics tools to assess whether rs78835907 or its linked genetic variants were associated with a putative function that might affect patient outcomes. HaploReg v2^21^ and the ENCODE^22^ were used to identify the regulatory potential of the region adjoining the SNPs. The GTEx data were used to identify the correlations between SNPs and prostate tissue-specific gene expression levels^23^. The publicly available cBioPortal for Cancer Genomics^24^ and MSKCC Prostate Oncogenome Project datasets^25^ were utilized in order to analyze *ATG16L1* gene expression, gene copy number, and clinical outcomes.

## Additional Information

**How to cite this article**: Huang, C.-Y. *et al.* Genetic variants of the autophagy pathway as prognostic indicators for prostate cancer. *Sci. Rep.* **5**, 14045; doi: 10.1038/srep14045 (2015).

## Supplementary Material

Supplementary Information

## Figures and Tables

**Table 1:** Clinical characteristics of study cohorts.

**Characteristic**		
**Localized prostate cancer cohort**	n (%)	*P*^a^
Patients, n	458	
Age at diagnosis		0.303
Median, y (IQR)	66 (61–70)	
≤65	211 (46.1)	
>65	247 (53.9)	
PSA at diagnosis		<0.001
Median, ng/mL (IQR)	11.1 (7.1–17.5)	
≤10	197 (44.9)	
>10	242 (55.1)	
Pathologic Gleason score, n (%)		<0.001
≤6	160 (35.3)	
>6	293 (64.7)	
Pathologic stage, n (%)		<0.001
T1/T2	303 (67.2)	
T3/T4/N1	148 (32.8)	
BCR	184 (40.2)	
Median follow-up time^b^, mo (95% CI)	54 (50–58)	
**Advanced prostate cancer cohort**	n (%)	*P*^c^
Patients, n	504	
Age at diagnosis		0.016
Median, y (IQR)	73 (66–79)	
≤72	250 (49.6)	
>72	254 (50.4)	
PSA at ADT initiation		0.022
Median, ng/mL (IQR)	33.8 (9.3–133.3)	
≤34	253 (51.6)	
>34	237 (48.4)	
Biopsy Gleason score at diagnosis, n (%)		<0.001
≤7	312 (63.4)	
>7	180 (36.6)	
Clinical stage at diagnosis, n (%)		<0.001
M0	308 (61.4)	
M1	194 (38.6)	
PSA nadir		<0.001
Median, ng/mL (IQR)	0.14 (0.01–1.06)	
<0.2	275 (54.8)	
≥0.2	227 (45.2)	
Time to PSA nadir		<0.001
Median, mo (IQR)	10 (5–20)	
<10	236 (47.0)	
≥10	266 (53.0)	
Treatment modality		0.002
ADT as primary treatment	254 (50.5)	
ADT for post RP PSA failure	73 (14.5)	
ADT for post RT PSA failure	12 (2.4)	
Neoadjuvant/adjuvant ADT with RT	122 (24.3)	
Others	42 (8.3)	
Disease progression	457 (90.7)	
Median follow-up time^b^, mo (95% CI)	87 (79–95)	

Abbreviations: IQR, interquartile range; PSA, prostate-specific antigen; BCR, biochemical recurrence; CI, confidence interval; ADT, androgen deprivation therapy; RP, radical prostatectomy; RT, radiation therapy.

^a^*P* value was calculated by the log-rank test for BCR in localized prostate cancer patients.

^b^Median follow-up time and 95% CIs were estimated with the reverse Kaplan-Meier method.

^c^*P* value was calculated by the log-rank test for disease progression in advanced prostate cancer patients.

**Table 2:** Association between SNPs in autophagy pathway and BCR in localized prostate cancer patients treated with RP.

**Gene SNP Genotype**	**Patients, n**	**Events, n**	**Median, mo**	**HR (95% CI)**^a^	***P***^a^	***q***
*ATG16L1* rs78835907						
GG	184 (41.7)	81 (45.8)	71	1.00		
GA	214 (48.5)	85 (48.0)	76	0.71 (0.51–0.98)	0.035	
AA	43 (9.8)	11 (6.2)	85	0.47 (0.24–0.91)	0.024	
GA/AA vs GG				0.67 (0.49–0.91)	0.012	**0.087**
AA vs GG/GA				0.56 (0.30–1.07)	0.082	0.167
Trend				0.70 (0.54–0.90)	0.006	**0.054**
*ATG16L1* rs13021297						
GG	214 (47.1)	72 (40.0)	121	1.00		
GA	196 (43.2)	86 (47.8)	71	1.59 (1.14–2.21)	0.006	
AA	44 (9.7)	22 (12.2)	53	1.71 (1.03–2.84)	0.039	
GA/AA vs GG				1.61 (1.17–2.21)	0.003	**0.054**
AA vs GG/GA				1.35 (0.84–2.16)	0.216	0.221
Trend				1.38 (1.10–1.72)	0.005	**0.054**
*MAP1LC3B* rs8044820						
AA	242 (54.5)	97 (54.8)	82	1.00		
AG	175 (39.4)	65 (36.7)	97	1.00 (0.73–1.39)	0.985	
GG	27 (6.1)	15 (8.5)	35	2.33 (1.31–4.16)	0.004	
AG/GG vs AA				1.12 (0.82–1.52)	0.473	0.245
GG vs AA/AG				2.33 (1.33–4.09)	0.003	**0.054**
Trend				1.24 (0.96–1.61)	0.101	0.167

Abbreviations: SNP, single nucleotide polymorphism; BCR, biochemical recurrence; RP, radical prostatectomy; HR, hazard ratio; CI, confidence interval; PSA, prostate-specific antigen.

^a^Adjusted by age, PSA at diagnosis, pathologic Gleason score, and pathologic stage. *q* < 0.1 are in boldface.

**Table 3:** Replication result of positive SNPs associated with disease progression in advanced prostate cancer patients treated with ADT.

**Gene SNP Genotype**	**Patients, n (%)**	**Events, n (%)**	**Median, mo**	**HR (95% CI)**^a^	***P***^a^
*ATG16L1* rs78835907					
GG	187 (37.3)	176 (38.8)	19	1.00	
GA	238 (47.5)	206 (45.4)	23	0.74 (0.60–0.91)	0.005
AA	76 (15.2)	72 (15.9)	16	0.95 (0.70–1.28)	0.721
GA/AA vs GG				0.78 (0.64–0.95)	**0.014**
AA vs GG/GA				1.14 (0.86–1.49)	0.365
Trend				0.91 (0.78–1.05)	0.193

Abbreviations: ADT, androgen deprivation therapy; HR, hazard ratio; CI, confidence interval; PSA, prostate-specific antigen.

^a^Adjusted by age, clinical stage, Gleason score, PSA at ADT initiation, PSA nadir, time to PSA nadir, and treatment modality. *P* < 0.05 are in boldface.
