# Supplementary material for: Genetic variants of the autophagy pathway as prognostic indicators for prostate cancer
Source: Sci Rep. 2015 Sep 14;5:14045. doi: 10.1038/srep14045 (PMC4568463; doi:10.1038/srep14045)
Supplement: Supplementary Information [file srep14045-s1.doc]

**Genetic variants of the autophagy pathway as prognostic indicators for prostate cancer**

Chao-Yuan Huang^1,†^, Shu-Pin Huang^2,3,†^, Victor C. Lin^4,5^, Chia-Cheng Yu^6,7,8^, Ta-Yuan Chang^9^, Te-Ling Lu^10^, Hung-Chih Chiang^10^ & Bo-Ying Bao^10,11,12,*^

^1^Department of Urology, National Taiwan University Hospital, College of Medicine, National Taiwan University, Taipei, Taiwan, ^2^Department of Urology, Kaohsiung Medical University Hospital, Kaohsiung, Taiwan, ^3^Department of Urology, Faculty of Medicine, College of Medicine, Kaohsiung Medical University, Kaohsiung, Taiwan, ^4^Department of Urology, E-Da Hospital, Kaohsiung, Taiwan, ^5^ School of Medicine for International Students, I-Shou University, Kaohsiung, Taiwan, ^6^Division of Urology, Department of Surgery, Kaohsiung Veterans General Hospital, Kaohsiung, Taiwan, ^7^Department of Urology, School of Medicine, National Yang-Ming University, Taipei, Taiwan, ^8^Department of Pharmacy, Tajen University, Pingtung, Taiwan, ^9^Department of Occupational Safety and Health, China Medical University, Taichung, Taiwan, ^10^Department of Pharmacy, China Medical University, Taichung, Taiwan, ^11^Sex Hormone Research Center, China Medical University Hospital, Taichung, Taiwan, ^12^Department of Nursing, Asia University, Taichung, Taiwan

^†^These authors contributed equally to this work.

^*^Correspondence and requests for materials should be addressed to B.Y.B. (bao@mail.cmu.edu.tw)

Supplementary Table S1 | Genotyped SNPs and the *P* values of their association with BCR after RP

| Gene | SNP ID | Chromosome | Position | BCR | | |
| --- | --- | --- | --- | --- | --- | --- |
|  |  |  |  | Additive | Dominant | Recessive |
| *ATG5* | rs4945747 | 6 | 106641630 | 0.208 | 0.219 | - |
| *ATG5* | **rs605457** | 6 | 106702245 | 0.370 | **0.046** | 0.248 |
| *ATG5* | rs547738 | 6 | 106738723 | 0.161 | 0.095 | 0.583 |
| *ATG5* | rs573775 | 6 | 106764866 | 0.343 | 0.659 | 0.201 |
| *ATG12* | rs26532 | 5 | 115173678 | 0.385 | 0.892 | 0.093 |
| *ATG12* | rs26537 | 5 | 115177014 | 0.767 | 0.335 | 0.276 |
| *ATG16L1* | **rs78835907** | 2 | 234119177 | **0.006** | **0.012** | 0.082 |
| *ATG16L1* | rs78765795 | 2 | 234120043 | 0.643 | 0.566 | 0.985 |
| *ATG16L1* | **rs13021297** | 2 | 234120552 | **0.005** | **0.003** | 0.216 |
| *ATG16L1* | rs12472651 | 2 | 234138522 | 0.414 | 0.656 | - |
| *ATG16L1* | rs10211468 | 2 | 234139467 | 0.578 | 0.347 | - |
| *ATG16L1* | rs7564252 | 2 | 234140610 | 0.401 | 0.273 | - |
| *ATG16L1* | rs3828309 | 2 | 234180410 | 0.051 | 0.057 | 0.256 |
| *ATG16L1* | rs3792106 | 2 | 234190740 | 0.294 | 0.144 | 0.999 |
| *ATG16L1* | **rs4663396** | 2 | 234192251 | 0.066 | **0.033** | - |
| *ATG16L1* | rs13005285 | 2 | 234194957 | 0.204 | 0.210 | 0.420 |
| *ATG16L1* | rs76072155 | 2 | 234203233 | 0.385 | 0.345 | 0.770 |
| *BECN1* | rs11552192 | 17 | 40962509 | 0.291 | 0.405 | 0.325 |
| *MAP1LC3B* | rs7204722 | 16 | 87430239 | 0.549 | 0.141 | 0.443 |
| *MAP1LC3B* | rs72806246 | 16 | 87434851 | 0.904 | 0.553 | 0.457 |
| *MAP1LC3B* | **rs8044820** | 16 | 87436382 | 0.101 | 0.473 | **0.003** |
| *SQSTM1* | **rs59066892** | 5 | 179234122 | **0.028** | 0.095 | - |
| *SQSTM1* | **rs58926938** | 5 | 179240712 | **0.049** | 0.173 | - |
| *SQSTM1* | rs10516140 | 5 | 179240910 | 0.849 | 0.650 | 0.695 |
| *SQSTM1* | **rs28495745** | 5 | 179243798 | **0.043** | 0.190 | **0.043** |
| *SQSTM1* | rs11747918 | 5 | 179245215 | 0.598 | 0.584 | 0.829 |
| *SQSTM1* | **rs1872779** | 5 | 179245749 | 0.108 | **0.033** | 0.693 |
| *SQSTM1* | rs502729 | 5 | 179248774 | 0.808 | 0.878 | - |
| *SQSTM1* | rs513165 | 5 | 179249073 | 0.467 | 0.807 | 0.348 |
| *SQSTM1* | rs67929139 | 5 | 179249179 | 0.642 | 0.596 | 0.822 |
| *SQSTM1* | rs78345236 | 5 | 179258176 | 0.096 | 0.250 | - |
| *SQSTM1* | rs77826446 | 5 | 179258184 | 0.289 | 0.323 | 0.514 |
| *SQSTM1* | rs2241349 | 5 | 179260009 | 0.503 | 0.732 | 0.379 |
| *SQSTM1* | rs2241350 | 5 | 179260478 | 0.132 | 0.138 | 0.426 |
| *SQSTM1* | rs10277 | 5 | 179264731 | 0.442 | 0.490 | - |
| *ULK1* | rs4964923 | 12 | 132388500 | 0.334 | 0.098 | 0.789 |
| *ULK1* | rs6598173 | 12 | 132388544 | 0.555 | 0.230 | 0.568 |
| *ULK1* | rs7295593 | 12 | 132395052 | 0.681 | 0.296 | 0.511 |
| *ULK1* | rs12303764 | 12 | 132399065 | 0.287 | 0.290 | - |
| *ULK1* | rs4964916 | 12 | 132405210 | 0.656 | 0.828 | - |

Abbreviations: SNP, single nucleotide polymorphism; BCR, biochemical recurrence; RP, radical prostatectomy; PSA, prostate-specific antigen.

*P* values were calculated using the multivariate Cox models adjusted for age, PSA at diagnosis, pathologic Gleason score, and pathologic stage.

*P* < 0.05 is in boldface.


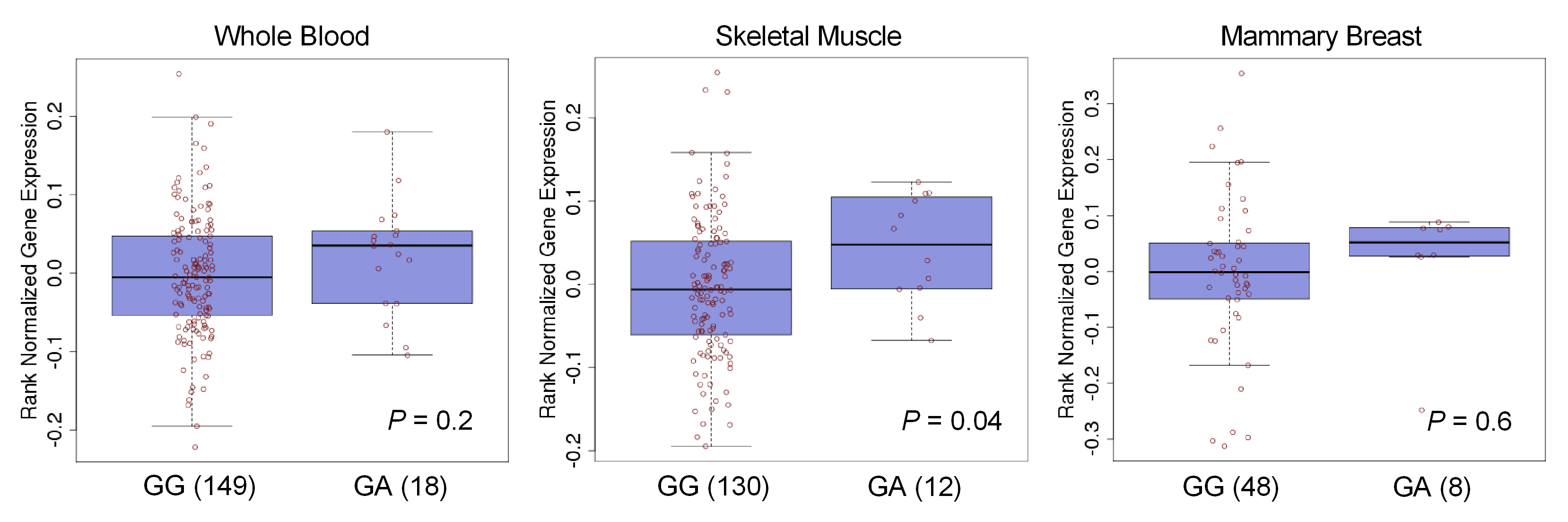


Supplementary Figure S1 | Expression quantitative trait locus association between rs78835907 genotype and *ATG16L1* expression in whole blood, skeletal muscle, and mammary breast tissues (GTEx data set). Numbers in parentheses indicate the number of cases.
